# Agreement with COVID-19 disinformation among Portuguese-speaking older adults: an international study

**DOI:** 10.1590/0034-7167-2023-0091

**Published:** 2023-08-11

**Authors:** Rodrigo Mota de Oliveira, Agostinho Antônio Cruz Araújo, Pricila Oliveira de Araújo, Anderson Reis de Sousa, Layze Braz de Oliveira, Inara Viviane de Oliveira Sena, Álvaro Francisco Lopes de Sousa, Isabel Amélia Costa Mendes

**Affiliations:** I Universidade de São Paulo. Ribeirão Preto, São Paulo, Brazil; II Universidade Estadual de Feira de Santana. Feira de Santana, Bahia, Brazil; III Universidade Federal da Bahia. Salvador, Bahia, Brazil; IV Universidade Federal do Piauí. Teresina, Piauí, Brazil; V Hospital Sírio-Libanês. São Paulo, São Paulo, Brazil

**Keywords:** Health Behavior, COVID-19, Disinformation, Aged, Global Health., Conductas Relacionadas con la Salud, COVID-19, Desinformación, Anciano, Salud Global, Comportamentos Relacionados com a Saúde, COVID-19, Desinformação, Idoso, Saúde Global.

## Abstract

**Objectives:**

to assess agreement with COVID-19 disinformation among Portuguese-speaking individuals aged 50 years or older.

**Methods:**

a descriptive and analytical study involving 1,214 older adults born in Portuguese-speaking countries. Data collection occurred through online information mining to recognize COVID-19 and disinformation content, and the application of a structured questionnaire.

**Results:**

agreement with disinformation content was 65.2%. Residing outside Brazil is a protective factor for agreement with disinformation content, and those who believe in the truthfulness of the information sources they receive were 31% more likely to agree with disinformation content.

**Conclusions:**

there is a high prevalence of disinformation among the older population in two Portuguese-speaking countries, which should raise the attention of healthcare professionals and guide coping strategies.

## RESUMO

**Objetivos:**

avaliar a concordância com conteúdos de desinformação relacionados à COVID-19 entre pessoas com 50 anos ou mais de idade, falantes do português.

**Métodos:**

estudo descritivo e analítico com 1.214 idosos nascidos em países de língua portuguesa. A coleta de dados ocorreu por meio da mineração de informações online para reconhecimento de conteúdos de desinformação e COVID-19, e aplicação de questionário estruturado.

**Resultados:**

a concordância com conteúdos de desinformação foi de 65,2%. Residir fora do Brasil é um fator de proteção para a concordância com conteúdos de desinformação, e aqueles que acreditam na veracidade das fontes de informação que recebem tiveram 31% mais probabilidade de concordar com conteúdos de desinformação.

**Conclusões:**

há alta prevalência de desinformação entre a população idosa em dois países de língua portuguesa, o que deve despertar a atenção dos profissionais da saúde e enfermagem, e orientar estratégias de enfrentamento.

## RESUMEN

**Objetivos:**

evaluar la concordancia con contenido de desinformación relacionado con COVID-19 entre personas de 50 años o más de edad, hablantes de portugués.

**Métodos:**

estudio descriptivo y analítico con 1.214 personas mayores nacidas en países de habla portuguesa. La recolección de datos se realizó mediante la minería de información en línea para el reconocimiento de contenido de desinformación y COVID-19, y la aplicación de un cuestionario estructurado.

**Resultados:**

la concordancia con contenido de desinformación fue del 65,2%. Residir fuera de Brasil es un factor de protección para la concordancia con contenido de desinformación, y aquellos que creen en la veracidad de las fuentes de información que reciben tuvieron un 31% más de probabilidades de estar de acuerdo con contenido de desinformación.

**Conclusiones:**

hay una alta prevalencia de desinformación entre la población mayor en dos países de habla portuguesa, lo que debe llamar la atención de los profesionales de la salud y la enfermería y orientar estrategias de enfrentamiento.

## INTRODUCTION

Autonomy for the performance of basic activities of daily living and self-care is of great value to human beings, but it is a condition that is generally affected in the elderly^(1)^. Circumstantial factors that involve the life of the elderly person encompass bio-psychosocial, mental, functional, and safety domains, and affect well-being, dignity, and autonomy^(2-3)^.

Active aging depends on the environment in which one lives, but, in principle, it depends on the individual when seeking functional and financial independence, social integration, and family support^(4)^. It is certain that the absence or fragility in one of these domains will entail vulnerabilities that need to be dimensioned, cared for, and supported. Sanitary emergencies add a series of consequences to the vulnerability of this population group, among which disinformation and susceptibility to inadvertent belief in narratives of close individuals or news dissemination vehicles stand out^(5-6)^.

In the COVID-19 pandemic, in addition to managing health services, countries and the entities that comprise them had to combat the spread of questionable information^(7)^. Therefore, the “infodemic” became a recurring term in discussions within the scope of global health^(8-9)^.

According to the World Health Organization (WHO), an infodemic is defined as the excess of false or misleading information available during a disease outbreak^(10)^. This phenomenon is detrimental to society, as it hinders the interpretation of information about a particular topic, especially in its judgment as true or false, which can lead to the adoption of health-risk behaviors. This context is further aggravated when considering the use of social media, as they amplify questionable content^(10-11)^.

In fact, disinformation crosses territorial borders, and it can be exacerbated when considering countries that share the same language^(11)^. Geographically, culturally, and religiously close countries that have the same or similar language share content that reinforces false news about the COVID-19 pandemic in their territories. A study conducted in countries where Portuguese is the main language revealed that, even with different populations, the content of disinformation about COVID-19 consumed in these regions was similar and accepted by a large part of the population^(11)^.

This statement is justified by considering the constant use of the internet, a resource often used to investigate one’s own health condition. As a result, personal access to information undermines trust in health services and promotes the adoption of behaviors that can cause individual or collective risks^(12-13)^. The new information technologies and the scope of social networks have not excluded older people from the digital world. However, older people are considered “digital immigrants” because they come from a generation that preceded the creation and availability of social media^(14)^, which can represent a barrier to understanding and handling technological tools. As a result of this context, this group has been identified as the most vulnerable in the adoption and dissemination of fake news, as they are not digital natives and have not been prepared for this new environment^(15)^.

With the facilities offered in the digital era, as well as the valorization of social networks, it is possible for any individual to be a creator and disseminator of content, in a rapid circulation of information, without any form of inspection or prior filtering^(16)^. Older people find information about their illnesses through various sources, mostly via the internet. For this generation, internet access was triggered in the workplace, via computer, and became available through mobile devices, as technology advanced in form, speed, and access to information^(17-18)^. When the interest and reason for the search are related to their illnesses, with the aim of better managing their treatment and gaining independence from information provided by doctors and other healthcare professionals, this population does not always feel secure with the information^(17,19)^.

There is literature pointing to the effect of disinformation on older people, suggesting that they are more vulnerable to disinformation compared to other younger age groups, and that older people not only have a decreased ability to correctly remember the source of the original information, but also demonstrate greater confidence in false memories, making them more vulnerable to disinformation^(20-21)^ and responsible for up to 80% of false news shared on social networks. However, there is a lack of studies that seek to deepen this consumption of disinformation and identify which factors are determining its occurrence in older people.

## OBJECTIVES

To evaluate agreement with COVID-19 disinformation content among Portuguese-speaking individuals aged 50 years or older.

## METHODS

### Ethical aspects

The study was conducted in accordance with the ethical research rules of the two participating countries, and was approved by the Brazilian Research Ethics Committee (CONEP) following the Declaration of Helsinki and relevant legislation in each country, including Resolution 466/12. Informed consent was obtained from all participants digitally.

### Study design

This is a cross-sectional, descriptive, and analytical study structured according to the STROBE guidelines.

### Study period

The research was conducted between June and August 2020, during the COVID-19 pandemic and the implementation of isolation and social distancing measures.

### Study location

The study included elderly individuals born in one of the seven Portuguese-speaking countries: Brazil, Portugal, Angola, Cape Verde, Guinea-Bissau, Mozambique, and São Tomé and Príncipe, but who were living in Portugal or Brazil for at least 3 months at the time of the survey.

### Population, sample, inclusion and exclusion criteria

To construct the sampling frame, participants were recruited using the snowball method adapted to the virtual environment. In the online adaptation of this method, the participant is responsible for recruiting other individuals from their own social network (minimum of 3 people). To ensure sample variability, we randomly selected 30 individuals in each of the two countries from a database of previous studies and called them “seeds.” These were purposely diversified with respect to the main confounding factors and biases in epidemiological studies: location in the country (divided according to the regions of each country); race/color (white and non-white); age group (50-60 years; 61 or older), educational level (primary/secondary education, higher education, and postgraduate education). Upon agreeing to participate, the first participants received the survey link and were instructed to invite/promote other similar individuals from their social network/community through the official invitation text and hyperlink sharing.

The total population of the study was 6843 individuals, of whom 1214 were 50 years of age or older and correctly completed all study questions, thus being eligible to participate in this study. To analyze the adequacy of the sample size, a power analysis was performed using G*Power 3.1 software, with post-hoc verification of the sample size required for contingency table statistics, considering a 95% confidence interval, alpha of 0.05, and an effect size of 0.15. The sample achieved a power of 99.8%, exceeding the minimum requirements for the sample. We chose to include individuals aged 50 or older, following recommendations from previous studies^(21-22)^ and considering them as a more or less uniform group that tends to exhibit a decline in cognitive function with age, which overloads their abilities related to cognitive functioning and abstract reasoning, as well as difficulties with digital technologies, limiting their abilities to differentiate between accurate and disinformation content^(21)^.

### Study Protocol

The main outcome of this study was agreement with at least one piece of disinformation content. To achieve this, responses of “agree” and “strongly agree” were grouped as “agreement.” As secondary variables, the social and demographic characteristics of the participants, their familiarity with COVID-19 (result of tests and knowing people who died due to the virus), consumption of content about the disease (sources of information), decision-making based on content, and willingness to vaccinate were evaluated^(11,23)^. Thus, the research was conducted in two phases:

1. Online data mining to identify the main subjects related to COVID-19 disinformation content discussed in Portuguese, as described in previous studies^(11,23)^.

For conceptual purposes and in accordance with relevant literature, the news was grouped into two categories: Category 1 - Conspiracy theories about the origin, prevention, treatment, and cure of COVID-19/SARS-CoV-2, and Category 2 - Home remedies and non-pharmacological methods for preventing contagion and treating SARS-CoV-2.

2. Online population survey with individuals from seven Portuguese-speaking countries (Brazil, Portugal, Angola, Cape Verde, Guinea-Bissau, Mozambique, São Tomé and Príncipe), but residents in Brazil or Portugal for more than 3 months.

The objective of this stage was to evaluate agreement with the content published on social networks studied and selected in phase 1^(11,23)^. A structured questionnaire was developed by the authors based on the literature^(24-26)^ and in two versions: Brazilian Portuguese and European Portuguese. This questionnaire contained 34 mostly multiple-choice questions and addressed social and demographic information (age, country of origin, nationality, religion, education, housing conditions), behaviors adopted to cope with the COVID-19 pandemic (social distancing, protective measures for COVID-19, and adherence to them), searching and consuming information and news about COVID-19, and 21 specific questions about agreement with the origin of SARS-CoV-2, prevention, treatment, and cure of COVID-19.

In this case, for every seven incorrect questions, one correct one was added as validation to avoid information biases. The questionnaire was evaluated by a group of 10 expert judges in the subject, five from each country, through two Delphi rounds to achieve consensus. The expert analysis covered the questionnaire’s construct validity and its cultural and linguistic properties.

The online questionnaire was hosted on a specific website that allowed for rapid data collection in both Brazilian and European Portuguese and allowed only one response per Internet Protocol (IP), i.e., one response per electronic device, thus avoiding multiple entries by the same user and, consequently, selection biases^(11)^.

### Analysis of Results and Statistics

The data were analyzed using the Statistical Package for the Social Sciences (SPSS) version 24.0 (SPSS Inc., Chicago, IL, USA). Descriptive analysis included absolute and relative frequencies. Prevalence ratios were used to evaluate crude associations (bivariate analysis), and their statistical significance was tested using Pearson’s chi-square test and the Monte Carlo method, considering a minimum significance level of p ≤ 0.05.

Ninety-five percent confidence intervals (CI 95%) were also established. Monte Carlo permutations were used to calculate p-values for independent variables with more than two categories of analysis, in order to obtain a better statistical fit of the data. All variables were previously analyzed to evaluate whether multicollinearity existed, based on tolerance coefficients and variance inflation factor (VIF) parameters.

Considering the high frequency of the reference outcome (agreement with COVID-19 disinformation greater than 10%), the association measure of traditional logistic regression analysis (odds ratio, OR) overestimates associations. Therefore, we opted for the Poisson regression model with robust variance estimation using a covariance matrix (generalized linear model) to estimate the prevalence ratio (PR), which is the most appropriate measure for cross-sectional studies. A logarithmic link function and 95% CI were used. The selection of variables for the multivariate model was based on the results of bivariate analyses, considering statistical significance (p-value ≤ 0.05), theoretical relevance, or better fit conditions. The observed parameters for the best performance adopted Akaike’s information criterion (AIC), log-likelihood, omnibus test, and effect tests (type III) as reference criteria.

## RESULTS

Of the 1214 study participants, 767 (63.2%) lived in Brazil, 123 (10.1%) were immigrants, and the age group of 50 to 59 years was the most prevalent (834; 68.7%), with 728 (60.0%) affirming a religious practice. In addition, 852 (70.2%) were in a relationship, with a higher prevalence of individuals with more than nine years of education (689; 56.8%). The agreement with disinformation content was 65.2% (792). The factors associated with this agreement are presented in Table 01.

**Table 1:** Association between agreement with **disinformation** content and social, demographic, and information source-related characteristics of study participants, 2022

Variables	Agreed with at least one disinformation content				*p* value
	Yes		No		
	n	%	n	%	
Immigrant					0.014
Yes	68	8.6	55	13	
No	724	91.4	367	87	
Age group					0.428
50 to 59 years old	538	67.9	296	70.1	
60 years or older	254	32.1	126	29.9	
Marital status					0.368
In a relationship	549	69.3	303	71.8	
Single	243	30.7	119	28.2	
Education					0.433
< than 12 years of study	131	16.8	59	14.0	
12 years or older	653	82.5	361	85.6	
Prefer not to answer	8	1	2	0.5	
Practice of any religion					0.000
Yes	558	70.5%	170	40.3	
No	234	29.5	252	59.7	
Do you agree with the need for social distancing/quarantine?					0.102
Agree	847	98.1	415	98.3	
Disagree	6	0.7	4	0.9	
Neutral (Neither agree nor disagree)	9	1.1	3	0.7	
"Do you agree with the strategies adopted by your local government to deal with the pandemic?"					0.178
Agree	593	74.9	322	76.3	
Disagree	188	23.7	87	20.7	
Neutral (Neither agree nor disagree)	11	1.4	13	3.1	
Have you tested yourself for COVID-19?	257	32.4	136	32.2	0.937
Do you usually receive and / or seek news about the infection by the new Coronavirus?	706	89.1	383	90.8	0.377
How often do you usually receive and/or seek news about the new Coronavirus infection?					0.182
Often	712	89.9	379	89.8	
Rarely	80	10.1	43	10.2	
Main sources of news and information					
TV shows	500	63.1	257	60.9	0.445
Online newspapers	566	71.5	324	76.8	0.046
Printed newspapers	74	9.3	26	6.2	0.055
Other sites	246	31.1	163	38.6	0.008
Social networks (Facebook, Twitter, Instagram)	358	45.2	179	42.4	0.352
WhatsApp	198	25.0	71	16.8	0.001
Friends and Family	193	24.4	103	24.4	0.988
Health professionals	422	53.3	235	55.7	0.423
Radio programs	144	18.2	62	14.7	0.123
In terms of trust, how do you classify the information from the sources that you listed as a priority?					0.000
Extremely reliable	517	65.3	308	71.8	
Some reliable and some not	275	34.7	119	28.2	

According to Table 02, living in a country other than Brazil was identified as a protective factor for agreement with disinformation content among elderly immigrants. In addition, those who believe in the veracity of the information sources they receive, regardless of the source, had a 31% higher likelihood of agreeing with disinformation content.

**Table 2:** Multivariate analysis of factors associated with agreement with **disinformation** content in elderly immigrants

Variable	PR	CI95%	aPR	CI95%	*p* value
Country of origin					0.02
Brazil	*Reference*				
Portugal	0.84	0.7 - 1	0.85	0.71 - 0.93	
African countries	0.74	0.59 - 0.93	0.76	0.60 - 0.96	
Education					
< than 12 years of study	*Reference*				
12 years or older	1.12	0.9 - 1.4			
Favorite news source					
Online newspapers	0.91	0.78 - 1.1			
Radio programs	1.08	0.9 - 1.3			
Printed newspapers	1.15	0.9 - 1.46			
TV shows	1.03	0.89 - 1.19			
Social media	1.05	0.91 - 1.21			
WhatsApp	1.16	0.99 - 1.37			
Friends and family	0.99	0.84 - 1.2			
Others	0.89	0.76 - 1.03			
Trust in preferred source					0.03
Depends on the source	*Reference*				
Reliable regardless of source	1.29	1.01 - 1.65	1.31	1.02 - 1.67	
Extremely reliable regardless of source	1.09	0.85 - 1.39	1.1	0.85 - 1.41	
Impact of the news received on their prevention attitudes					
No impact	*Reference*				
Low impact	1.13	0.72 - 1.77			
Moderate impact	1.03	0.68 - 1.58			
High impact	1.04	0.68 - 1.59			
Frequency with which you receive news about COVID-19 sent by others					
Daily	*Reference*				
More than once a week	0.97		0.8 - 1.17		
Weekly	0.98		0.77 - 1.24		
Rarely	0.93		0.77 - 1.12		

## DISCUSSION

Our findings point to the high prevalence of disinformation among the elderly population in the context of the COVID-19 pandemic in two Portuguese-speaking countries. Although this finding is alarming, it has been repeated in other health crisis contexts^(27-29)^. For example, prior to the COVID-19 pandemic, the impact of disinformation among the elderly population was evidenced in different health and disease situations, such as sexual practices and sexually transmitted infections, as well as immunization, with indications for overcoming the deconstruction of prejudiced and inhibiting ideas regarding the broader understanding of the issue.

The findings among the elderly population are in line with studies focused on the general population conducted in other contexts, as there are studies indicating a high prevalence of agreement with disinformation about COVID-19 (63.9%) in different age groups, values close to those found in this study^(11,30)^. However, there are peculiarities in the case of the elderly that must be considered.

There are several factors that can influence the higher consumption of false information about COVID-19 in the elderly^(31-32)^. Lack of digital and health literacy are two important factors that must be considered. Elderly people who are not familiar with the internet or social media may have difficulty navigating online information sources and using fact-checking tools, making them more susceptible. In addition, older individuals with limited health literacy may have difficulty understanding medical information, even when it is transmitted through digital media, and may be more vulnerable to false claims about treatments or prevention strategies for emerging diseases such as COVID-19^(33-34)^.

Older people become easier targets for disinformation because they are from a time when access to information was provided through newspapers and magazines, books, and encyclopedias that generally went through treatment and verification, which brought the feeling of correct and reliable information. In contrast, the COVID-19 infodemic hit people abruptly who are unfamiliar with or do not have the skills to use technology and the internet and critically assess information and its sources^(34)^. Traditionally, this issue tends to have a greater impact on those elderly people with lower levels of education and income, due to the sum of vulnerabilities^(35-36)^. However, in our study, the participants mostly had high education and income levels, which differs from previous findings. This can be explained, in part, by the influence of salutogenic/healthism, a kind of belief system in which individuals see themselves as primarily or solely responsible for their health, in people with higher education, leading them to have greater distrust of medical authorities and conventional medicine, making them more prone to resort to “alternative truths” and explanations that lack theoretical or empirical support^(11,37)^.

Confirmation bias, when individuals seek information that confirms their existing beliefs or biases, is also more present in older people, causing them to have certain beliefs or opinions that lead them to have a higher probability of consuming information that confirms these beliefs, even if they are not accurate^(32,38)^. In addition, the elderly may be more likely to trust information from their close social networks, which may include friends and family, who are also susceptible to disinformation, further amplifying the dissemination of erroneous information within this age group.

In our study, it became evident that not only is the information itself decisive for disinformation, but also the source of the information and who propagates it. Traditionally, people rely on information sources from classic news agencies or credible websites to stay informed about the most current events. However, when these sources are perceived as biased, untrustworthy, or present content that is very different from what the subjects want to perceive as truth, individuals may be more likely to seek alternative sources of information, including those that promote conspiracy theories or false and biased narratives, as observed in our results^(11,39)^.

There is a series of studies^(11,39-41)^ showing that individuals who have social media as their main source of news are more likely to be exposed to false news, as these platforms generally amplify sensational or deceptive content. In addition, individuals who have a strong ideological or partisan identity may be more likely to consume false news that aligns with their existing beliefs, even if they are not accurate. For example, in our study, giving credibility to the truthfulness of the information they receive, regardless of the source, increased the likelihood of agreeing with disinformation content, reinforcing the impact of the source and the sender on the consumption of disinformation. It is extremely important that people critique the information sources they consume and seek reliable sources that prioritize accuracy and objectivity. Media literacy programs can help in this process, developing in people, especially older ones, the necessary skills to distinguish between real and deceptive news and use fact-checking mechanisms.

Governments and leaders play a crucial role in combating disinformation about COVID-19, ensuring that accurate information about this pandemic is easily accessible to the public through various channels, working with fact-checking organizations to identify and correct incorrect information, regulating the spread of disinformation about COVID-19 on social media and other online platforms, and collaborating with international organizations and other countries to share accurate information^(11,42)^.

In our study, it became clear that being from another country, other than Brazil, is a protective factor for agreement with disinformation content among older adults. Although it is not possible to assert that Brazilian seniors consume more disinformation content about Covid-19 than those in other Portuguese-speaking countries, there is evidence in the literature that suggests that political factors played a role in shaping public perceptions about the pandemic in the country^(11,43)^.

Brazil was one of the countries hardest hit by the pandemic studied, with more than 14 million confirmed cases and more than 380,000 deaths in March 2023^(44)^. The country’s response to the pandemic was highly politicized, with people in high management positions minimizing the severity of the virus and resisting public health measures such as social isolation and mask-wearing. Additionally, the crisis situation due to the pandemic in Brazil was long-lasting and critical, leading Brazilians to spend a lot of time in social isolation. Socially isolated elderly people may have limited access to accurate information and are more likely to rely on unreliable sources of information^(45-46)^.

In contrast, in the Portuguese context, a population survey revealed that 7 in 10 Portuguese people are concerned about what is real or false on the internet. However, the percentage of Portuguese people denying such concern decreased by 2.9 percentage points in the subsequent survey^(47)^.

Thus, the issue of disinformation consumption related to COVID-19 among older adults should draw the attention of nursing and healthcare teams, regarding behavioral factors and the influence of high-impact phenomena on the configuration of habits, attitudes, and practices intertwined with the process of human aging. It is important to emphasize that it is necessary to understand in a broad sense the reasons for the high prevalence of agreement with disinformation among elderly people, in order to avoid reinforcing ageist ideas^(48)^ that strengthen the stigmatization of aging in the face of the establishment of prohibitive and non-educational measures, making the elderly person the “other” of the pandemic^(49)^. In this sense, space must be opened for the knowledge of variables such as the level of education of the elderly, policies for accessing information available in and between countries, the absence of regulatory frameworks for direct and daily access to news, religiosity, and the rise of ideologically negationist movements.

### Limitations

This research presents important limitations. As a cross-sectional study, it is not possible to establish a cause-and-effect relationship. Another limiting aspect was that data collection was conducted online with elderly people from countries with significant socioeconomic differences, which possibly led to a heterogeneous study population.

### Contributions to the field of nursing and public health

As contributions to the field of health and nursing, the results of this study highlight the need to disseminate knowledge about the harms of disinformation among elderly people. It’s imperative to share ways to identify and dealing with it, to acknowledge the doubts, fears, anxieties, and concerns of elderly people regarding the use of technology and the consumption of news on the internet, health professionals should recognize the vulnerability of elderly people while also acknowledging their capacity for learning and questioning the legitimacy of news. Additionally, health professionals should adopt self-vigilant stances in combating age-related stereotypes about internet use. Finally, developing and implementing public policies that are capable of including the elderly population while respecting their rights, autonomy, and potentialities is crucial.

## CONCLUSIONS

There is a concerning scenario of high agreement with disinformation related to COVID-19 among Brazilian and Portuguese elderly people. The two main variables that influence this agreement are the country of birth and the credibility given to the news source. It is important to provide elderly people with accurate and reliable sources of information on COVID-19 and help them develop the necessary skills to navigate online information sources and avoid false information. Health literacy programs can help people develop the skills needed to distinguish between real and fake news and identify sources of bias or disinformation.
